# The Effects of Laser Moxibustion on Knee Osteoarthritis Pain in Rats

**DOI:** 10.1089/photob.2019.4716

**Published:** 2020-01-09

**Authors:** Yuan Li, Fan Wu, Jianzi Wei, Lixing Lao, Xueyong Shen

**Affiliations:** ^1^ School of Acupuncture, Moxibustion and Tuina, Shanghai University of Traditional Chinese Medicine, Shanghai, China.; ^2^ School of Chinese Medicine, The University of Hong Kong, Hong Kong, SAR, China.; ^3^ Shanghai Research Center of Acupuncture & Meridian, Shanghai, China.

**Keywords:** laser moxibustion, knee osteoarthritis, chronic pain

## Abstract

***Background:*** Although chronic pain affects the quality of life of patients with osteoarthritis, current medical treatments are either ineffective or have long-term side effects. Recently, low-intensity laser irradiation of corresponding acupoints was demonstrated to alleviate pain.

***Objective:*** The aim of the present study was to investigate the effects of 10.6 μm laser moxibustion on a monosodium iodoacetate (MIA)-induced knee osteoarthritis pain model.

***Methods:*** Thirty-two rats were randomly assigned to four groups: Saline, MIA, MIA+Laser, and MIA+Sham Laser. The 10.6 μm laser was used to irradiate the ST35 for 10 min once a day for a total of seven applications. The paw withdrawal mechanical threshold and weight-bearing difference were performed to evaluate the analgesic effects of laser moxibustion. At the end of the experiment on days 28, the joint histology, the levels of metalloproteinases-13 (MMP-13) in the cartilage, and TNF-α, IL-1β, and IL-6 in the synovial membrane were measured to determine the chondroprotection and anti-inflammatory effect of laser moxibustion.

***Results:*** Early laser moxibustion significantly reversed the MIA-induced mechanical hyperalgesia and weight-bearing difference, especially on the 28th day (*p* < 0.001). Moreover, laser moxibustion prevented the articular pathological lesions and cartilage destruction on days 28 (*p* < 0.01). Remarkably, the levels of cartilage MMP-13, and synovial TNF-α, IL-1β, and IL-6 also decreased on day 28 (*p* < 0.05) after the early treatment of laser moxibustion.

***Conclusions:*** 10.6 μm laser moxibustion may have long-lasting analgesic, anti-inflammatory, and chondroprotection effects, suggesting that it may emerge as a potential therapeutic strategy for the chronic pain treatment of osteoarthritis.

## Introduction

Knee osteoarthritis (KOA) is a degenerative disease, including synovitis, articular cartilage destruction, subchondral bone reconstruction, and other pathological changes, accompanied by persistent pain and joint dysfunction, greatly affecting the quality of life of patients.^1^ However, the mechanism of chronic pain in KOA remains unclear. In recent years, animal models simulating the pathological changes of human KOA have provided a broad space for the study of the molecular mechanism of KOA.^2^ The intra-articular injection of monosodium iodoacetate (MIA), a commonly used chemical KOA model, results in the histological changes of articular cartilage and especially, a nociceptive response similar to the pathology and pain sensation observed in patients with KOA.^3,4^

There are studies on the structural changes of KOA induced by MIA, and pain mechanisms are limited to the local joint at the peripheral level,^5^ involving inflammatory mediators, structural proteins (extracellular matrix hydrolase), and cell signaling pathway-related protein kinases-mediated synovitis and chondrocyte apoptosis.^6–8^ Synovial inflammation of the knee joint induces the infiltration of immune cells and secretes a large number of inflammatory mediators, such as proinflammatory cytokines (TNF-α, IL-1β, and IL-6), which further increase the expression of matrix metalloproteinases (MMPs), promote the catabolism of chondrocyte, and aggravate the degradation of extracellular matrix.^9^

For the treatment of KOA, nonsteroidal anti-inflammatory drugs are primarily used for acute pain, but are not particularly effective for chronic pain.^10,11^ Nondrug therapies, such as acupuncture, moxibustion, low-intensity laser, proper exercise, and weight control, can also alleviate pain and improve articular function to varying degrees, but the exact therapeutic mechanism has not been clarified.^12–14^

Laser acupuncture, a product combining modern laser, traditional acupuncture and moxibustion, has been used in the treatment of inflammatory pain, and its curative effect is remarkable.^13,15,16^ The low-intensity laser irradiation of corresponding acupoints was also demonstrated to significantly alleviate the pain of patients with KOA.^13,15,16^ A convincing study suggested that 10.6 μm laser moxibustion has a good thermal therapeutic effect similar to moxibustion while avoiding the shortcomings of traditional moxibustion-induced smoke pollution and compensates for the limited warming effect of common laser needles currently used in clinical settings.^17,18^ However, the underlying mechanisms of its effects on chronic joint pain are still not completely understood. Our preliminary clinical trials have shown that laser moxibustion at a 10.6 μm wavelength significantly improved joint pain and articular dysfunction in patients with KOA.^19,20^

To further determine the analgesic and cartilage protective effects of 10.6 μm laser moxibustion on KOA, we investigated the effect of 10.6 μm laser moxibustion on pain hypersensitivity and articular cartilage destruction in MIA-induced KOA model.

## Materials and Methods

### Animals and ethics

Male Sprague–Dawley rats (220–250 g, SCXK2013-0016; Xipuer-Bikey Co., Ltd., Shanghai, China) were housed in a controlled condition (22°C–24°C, relative humidity 40–60%, and 12:12 h light:dark cycle) with food and water ad libitum. All experimental procedures were approved by the Shanghai University of Traditional Chinese Medicine Animal Welfare and Ethics Committee (PZSHUTCM18113003) and conformed to the standards of the International Council for Laboratory Animal Science.

### MIA-induced KOA

Animals (*n* = 32) were distributed randomly into four groups: Saline, MIA, MIA+Laser, and MIA+Sham Laser (*n* = 8 per group). Rats in the MIA, MIA+Laser, and MIA+Sham Laser groups were anesthetized with isoflurane and subject to a single intra-articular injection of MIA (3 mg/50 μL; Sigma) dissolved in 0.9% saline in the left knee joint through the infrapatellar ligament, as previously described.^21^ The MIA+Laser and MIA+Sham Laser groups received intervention procedures. The saline group only received 50 μL of 0.9% saline into the left knee joint without any treatment.

### Laser moxibustion treatment

Laser moxibustion treatment was applied using a 10.6 μm laser device (SX10-C1; Wonderful-Opto-Electrics Tech Co, Ltd., Shanghai, China) as previously described.^21,22^ One day after the MIA injection, rats were immobilized on the platform by the operator. Rats in the MIA+Laser group were exposed to laser irradiation, the parameters are presented in Table 1, at the ST-35 acupoint located in the depression of the lateral aspect of the infrapatellar ligament for seven consecutive days (once per day).^23^

**Table 1.** The Parameters of Laser Moxibustion Device

Wavelength, μm	Irradiance, mW/cm^2^	Energy density, J/cm^2^	Each treatment duration, min	Treatment frequency	Cumulative dose, J
10.6	80	1500	10	One time per day	329.86

For the MIA+Sham Laser group, the rats were subjected to the same procedure as the Laser group, but without laser output and with only the indicator lights on.

### Behavioral tests

All rats were acclimated to the test environment for 30 min. The paw withdrawal threshold was measured using an Electronic Von Frey Aesthesiometer (IITC Life Science). The rigid probe was perpendicularly applied to the plantar surface of the ipsilateral hind paw with increasing force until paw withdrawal or a flinching response. The average of 3 sec-time was recorded as the result of paw withdrawal mechanical threshold (PWMT) for each rat.^24^ The weight-bearing difference was tested using an Incapacitance Tester (MR-600; IITC Life Science). Rats were placed in a plexiglass chamber and each paw positioned separately on a load-bearing plate. The percentage of ipsilateral weight born was calculated according to the following: [(the weight distribution of the ipsilateral hind paw/the weight distribution of the ipsilateral plus contralateral hind paw) × 100%]. The mean value of 3 sec-time was determined as the result of the weight born for each rat.^21^ The operator was blinded to grouping.

### Joint histology

Rats were deeply anesthetized 28 days after the behavioral tests and transcardially perfused with 0.9% saline followed by 4% paraformaldehyde. Each left knee joint was resected, fixed in 10% formalin (Sigma-Aldrich) at 4°C for 48 h, decalcified with ethylenediaminetetraacetic acid decalcifying solution (Solarbio) for 4 weeks, and then embedded in paraffin. The coronal sections (7 μm) were stained with hematoxylin and eosin (HE) and Safranin O (SO)-fast green, and then observed using a microscope. Cartilage degeneration was assessed blindly using the Osteoarthritis Research Society International (OARSI) score, which combined the score of grade and stage (0–24).^25^

### Immunohistochemical staining

The immunohistochemistry sections were deparaffinized, rehydrated, and blocked with normal goat serum. The sections were incubated with antibodies to MMP-3 (1:400, ab39012; Abcam) at 4°C overnight, followed by horseradish peroxidase-conjugated secondary antibody (1:200, ab97051; Abcam). The slides were visualized using 3,3-diaminobenzoic acid and counterstained with hematoxylin. Finally, the sections were mounted with a coverslip and Canada balsam. For the quantitative analysis of immunohistochemical staining, the immunoreactivity was determined through the average optical density (AOD) in the fields obtained from three sections for each rat using Image Pro plus 6.0 software. The operator was blinded to treatment.

### Enzyme-linked immunosorbent assay

The synovial membrane was collected and centrifuged at 14,000 r/min at 4°C for 10 min, and the supernatants were then extracted for the measurement of enzyme-linked immunosorbent assay (ELISA). The concentrations of TNF-α, IL-1β, and IL-6 were analyzed using ELISA kits according to the manufacturer's protocol.

### Statistical analysis

Data are presented as the mean ± standard deviation and analyzed using GraphPad Prism7.0 software. The behavioral tests were analyzed by two-way ANOVA for repeated measurement followed by Bonferroni's post hoc test. The OARSI score was analyzed by Kruskal–Wallis test followed by Dunn's post hoc test among the groups. The values of MMP-13, TNF-α, IL-1β, and IL-6 were conducted by one-way ANOVA followed by Tukey's post hoc test for multiple comparisons. *p* < 0.05 were considered statistically significant.

## Results

### Laser moxibustion reversed MIA-induced pain behavior

**FIG. 1.**
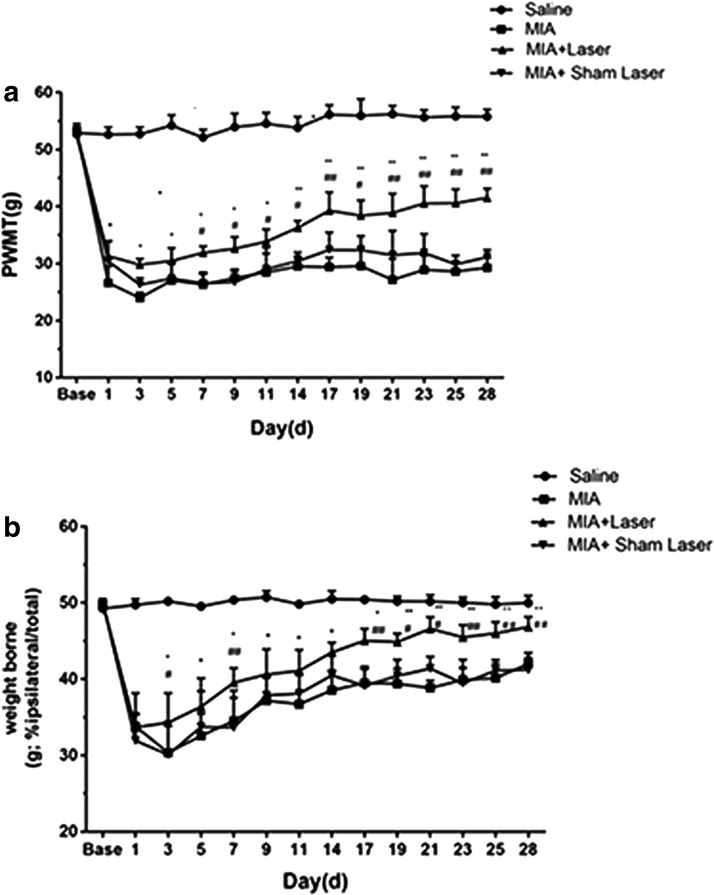
Effects of laser moxibustion **(a)** on MIA-induced mechanical hyperalgesia and **(b)** weight-bearing asymmetry. Data are presented as mean ± SD (*n* = 8) and analyzed using two-way ANOVA for repeated measurement. *p < 0.05 and **p < 0.001 compared to MIA group; ^#^*p* < 0.05 and ^##^*p* < 0.001 compared to MIA+Sham Laser group. MIA, monosodium iodoacetate; SD, standard deviation.

As expected, the injection of MIA presented decreased mechanical hypersensitivity threshold and weight-bearing difference in the ipsilateral hind paw for up to 28 day. Compared with the MIA and/MIA+Sham Laser groups, seven consecutive days of treatment laser moxibustion at the ST35 increased the PWMT and alleviated the weight-bearing difference in the ipsilateral hind paw of KOA rats (*p* < 0.001). Remarkably, on the 17th to 28th day after treatment, the analgesic effect of laser moxibustion on the later stage of pain was obvious, thus suggesting that laser moxibustion may have a certain cumulative therapeutic effect on MIA-induced KOA pain (Fig. 1a, b).

### Laser moxibustion prevented articular degradation

**FIG. 2.**
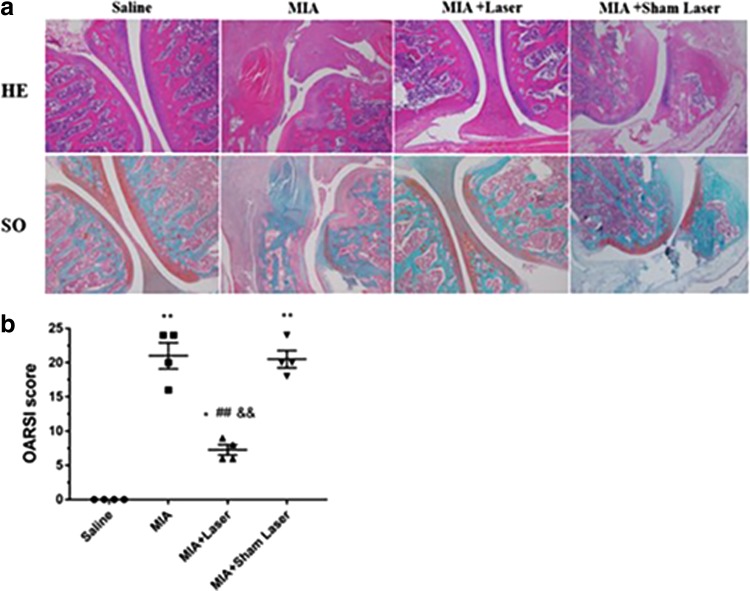
Effects of laser moxibustion on MIA-induced articular pathological degeneration. **(a)** The joint histology image of HE and SO staining (40 × ). **(b)** Histopathologic assessment of cartilage classified using the OARSI scoring system. Data are presented as mean ± SD (*n* = 4) and analyzed using Kruskal–Wallis test. *p < 0.01 and **p < 0.001 compared to saline group; ^##^*p* < 0.001 compared to MIA group; ^&&^*p* < 0.001 compared to MIA+Sham Laser group. HE, hematoxylin and eosin; OARSI, Osteoarthritis Research Society International; SO, Safranin O.

According to the HE and SO staining, both the MIA and MIA+Sham Laser groups exhibited severe cartilage damage and proteoglycan loss 28 days after MIA injection compared with the Saline group. Laser moxibustion significantly inhibited cartilage destruction and proteoglycan loss in MIA-induced KOA rats (Fig. 2a). Consistent with the results of joint staining, a significantly elevated OARSI score was observed in the MIA and MIA+Sham Laser groups compared to the saline group and MIA+Laser group (*p* < 0.001), while the decreased OARSI score in the MIA+Laser group (Fig. 2b) was observed compared to the MIA and MIA+Sham Laser groups (*p* < 0.01), but its level remained higher than the saline group (*p* < 0.01), indicating that laser moxibustion may have an chondroprotection effect on MIA-induced articular degradation.

### Laser moxibustion reduced MMP-13 in the cartilage of MIA-induced KOA

**FIG. 3.**
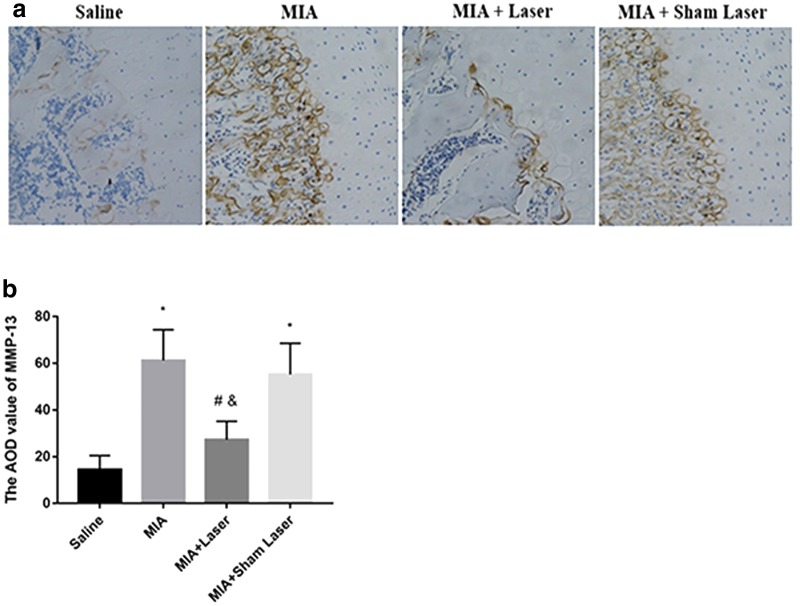
Effects of laser moxibustion on the expression of MMP-13 in MIA-induced KOA rats. **(a)** Immunohistochemical staining was used to identify the expression of MMP-13 in the articular cartilage (200 × ). **(b)** Histogram of the AOD value of MMP-13 expression among the four groups. Data are presented as mean ± SD (*n* = 4) and analyzed using one-way ANOVA. *p < 0.01 compared to saline group; ^#^*p* < 0.01 compared to MIA group; ^&^*p* < 0.01 compared to MIA+Sham Laser group. AOD, average optical density; KOA, knee osteoarthritis; MMP-13, metalloproteinases-13.

The expression of MMP-13 in the cartilage was observed through immunohistochemical staining (Fig. 3a). The AOD value of MMP-13 was significantly higher in the MIA and MIA+Sham Laser groups than that in the Saline and MIA+Laser groups (*p* < 0.01). Furthermore, the AOD value of MMP-13 was significantly decreased in laser groups compared with the MIA and MIA+Sham Laser groups (*p* < 0.01), while with no statistical difference compared with saline group (*p* > 0.05). Thus, laser moxibustion treatment inhibited the MMP-13 expression in cartilage of MIA-induced KOA (Fig. 3b).

### Laser moxibustion decreased the synovial levels of TNF-α, IL-1β, and IL-6 in MIA-induced KOA

**FIG. 4.**
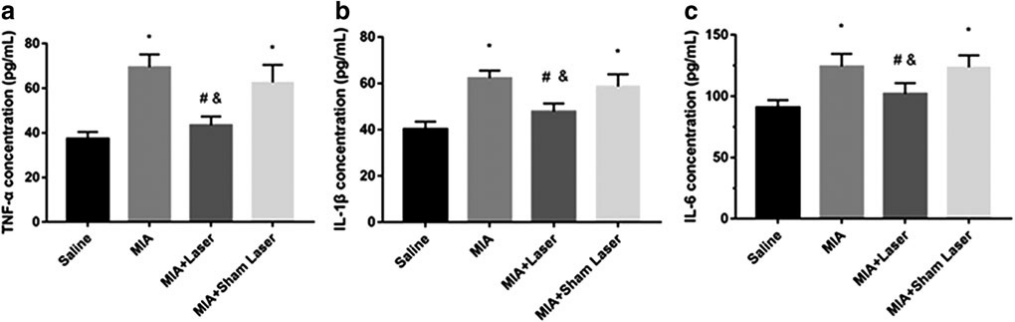
Effects of laser moxibustion on the level of TNF-α, IL-1β, and IL-6 in the synovium of MIA-induced KOA rats. The concentrations of **(a)** TNF-α, **(b)** IL-1β, and **(c)** IL-6 in the synovium were measured in four groups. Data are presented as mean ± SD (*n* = 4) and analyzed using one-way ANOVA. *p < 0.05 compared to saline group; ^#^*p* < 0.05 compared to MIA group; ^&^*p* < 0.05 compared to MIA+Sham Laser group.

As presented in Fig. 4a–c, the concentrations of TNF-α, IL-1β, and IL-6 in the MIA and MIA+Sham Laser groups were markedly higher than the Saline group and MIA+Laser group (*p* < 0.05). However, the group treated with laser moxibustion had decreased levels of TNF-α, IL-1β, and IL-6 compared with the MIA and MIA+Sham Laser groups (*p* < 0.05). There was no statistical difference in the level of these cytokines between the saline and MIA+Laser group (*p* > 0.05). The results suggested that laser moxibustion reduced the expression of proinflammatory cytokines in the MIA-induced KOA model.

## Discussion

Acupuncture and moxibustion has been well demonstrated to relieve joint pain and improves articular dysfunction.^12,26^ However, the adverse effects of moxa smoke restrict the development of traditional moxibustion.^27,28^ In recent years, our group has developed a 10.6 μm laser moxibustion apparatus. The infrared radiation spectrum of traditional indirect moxibustion and human acupoints are very similar, and their radiation peaks are about 10 μm, which may produce resonance, playing a therapeutic role. In this wavelength, the infrared light is easily absorbed by skin, producing a good thermal effect similar to moxibustion.^17,18^ Several clinical trials have shown that low-intensity laser irradiation of corresponding acupoints can significantly alleviate the pain of patients with KOA, promote joint function, and improve the quality of life.^13,15,16^ Basic experimental studies have found that a low-intensity laser can inhibit inflammatory cell infiltration, reduce the expression of proinflammatory cytokines and produce antinociceptive effects.^29^ Histopathology showed that a low-intensity laser could alleviate synovitis, cartilage injury, and subchondral bone destruction, which may have a certain cartilage protection effect.^30^ However, most of the currently used low-intensity lasers are semiconductor lasers, and more importantly, they only have the effect of a “light needle” due to their limited thermal effects.^31^ Therefore, they have no significant effect on the treatment of chronic pain. Convincing evidence demonstrated that our 10.6 μm laser moxibustion has a long-lasting thermal effect and also affects deep tissues,^32^ which may provide a therapeutic strategy for the clinical treatment of osteoarthritis pain.

MIA is a dose- and time-dependent KOA model. High doses of MIA can induce persistent inflammation, degenerative changes, and chronic pain.^3^ The PWMT and weight-bearing difference are the main tests to evaluate the pain behavior of the KOA pain model.^33^ Consistent with other studies, we found that high doses of MIA can induce persistent hyperalgesia and weight-bearing differences. Between day 1 and 7 post-MIA injection was the acute inflammation stage, which caused acute pain. After 14 days, the inflammation was greatly resolved and entered into the stage of chronic pain. Therefore, we applied laser moxibustion in the early time to investigate the role of laser moxibustion on the procession of acute inflammation to chronic pain in the MIA-induced KOA model. The results showed that early laser moxibustion treatment could significantly reverse the PWMT and weight-bearing difference induced by MIA, especially in the later stage of KOA, indicating that laser moxibustion might have a long-lasting antinociceptive effect of MIA-induced chronic pain.

The generation of inflammatory mediators and degradation of the extracellular matrix by MIA-injection are crucial causes for articular cartilage destruction.^25^ In the present study, early laser moxibustion markedly inhibited the destruction of articular cartilage and matrix degradation, and decreased the OARSI score. Furthermore, early laser moxibustion inhibited the increased expression of TNF-α, IL-1β, IL-6, and MMP-13 in articular cartilage post-MIA injection. Although inflammation gradually dissolved on 14 days, proinflammatory cytokines were still produced, suggesting that MIA-induced KOA models were accompanied by low levels of persistent inflammation during its procession. Overall, our results suggested that the antinociceptive action of 10.6 μm laser might occur by inhibiting synovial inflammation, reducing extracellular matrix catabolism, and preventing cartilage damage, which may underlie the long-lasting analgesia effect mediated by laser moxibustion at the peripheral level.

Chronic pain resulting from osteoarthritis is a serious public health issue. One of the main challenges in chronic pain research is to develop new therapies that are effective for long-term and have fewer side effects. Clinical trials and basic studies have shown that nondrug therapies, such as acupuncture, moxibustion, and low-intensity laser therapy, are important alternative therapies for relieving KOA joint pain and improving joint dysfunction. In this study, we found that 10.6 μm laser moxibustion applied in the early stage of MIA-induced KOA significantly inhibit chronic joint pain and joint pathological damage by decreasing levels of TNF-α, IL-1β, IL-6, and MMP-13 in cartilage, which not only induced the chondroprotection effect but also maintained a long-lasting analgesic and anti-inflammatory effect. Another study showed that 840 nm light-emitting diode irradiation for a week after 7 days of MIA-induced KOA prevented cartilage damage and subchondral bone destruction, and significantly reduced inflammatory cell infiltration and pannus formation.^34^ Moreover, Uryu et al. used traditional moxibustion on an injured knee joint and found that there were no antinociceptive effects of moxibustion after 7 days of treatments, while continued moxibustion treatments significantly increased the weight born at day 14 and lasted until the end of day 28.^35^ Ma et al. also determined that early electroacupuncture (EA) intervention can significantly reduce joint pain and pathological lesions induced by MIA injection compared with mid- and late-term EA interventions.^36^ Interestingly, a study reported that a 808 nm near infrared laser generated the antianalgesic effect from 5 to 30 min after treatment and vanished after 60 min.^37^ Kim and Kim conducted a 3-week treatment using an 850 nm gallium-aluminum-arsenide laser, resulting in the weight-bearing difference, PWMT, and PWL significantly reversed, and the serum levels of TNF-α, IL-1β, and IL-6 also decreased in a MIA-induced KOA model.^29^ These results indicated that different parameters of laser intervention have different effects on MIA-induced KOA pain. Future studies should compare the parameters of laser, determining the optimal treatment parameters, producing long-lasting analgesic effects, and more importantly, reducing or replacing the use of drugs.

## Conclusions and Summary

This study demonstrated the early intervention of laser moxibustion irradiated at ST35 significantly reversed the PWMT and weight-bearing difference in MIA-induced KOA pain rat. The joint histology showed that 10.6 μm laser moxibustion inhibited the procession of cartilage damage and decreased the OARSI score. Moreover, the levels of cartilage MMP-13 and synovial TNF-α, IL-1β, and IL-6 were also reduced by the early laser moxibustion treatment. These results suggested that 10.6 μm laser moxibustion may play an important role in pain relief and chondroprotection in MIA-induced KOA model.
